# Developing a Modern Greenhouse Scientific Research Facility—A Case Study

**DOI:** 10.3390/s21082575

**Published:** 2021-04-07

**Authors:** Davor Cafuta, Ivica Dodig, Ivan Cesar, Tin Kramberger

**Affiliations:** 1 Department of Information Technology and Computing, Zagreb University of Applied Sciences, 10000 Zagreb, Croatia; ivan.cesar@tvz.hr (I.C.); tin.kramberger@tvz.hr (T.K.); 2 Multimedia, Design and Application Department, University North, 42000 Varaždin, Croatia

**Keywords:** internet of things, artificial intelligence, sensors smart agriculture, cloud computing

## Abstract

Multidisciplinary approaches in science are still rare, especially in completely different fields such as agronomy science and computer science. We aim to create a state-of-the-art floating ebb and flow system greenhouse that can be used in future scientific experiments. The objective is to create a self-sufficient greenhouse with sensors, cloud connectivity, and artificial intelligence for real-time data processing and decision making. We investigated various approaches and proposed an optimal solution that can be used in much future research on plant growth in floating ebb and flow systems. A novel microclimate pocket-detection solution is proposed using an automatically guided suspended platform sensor system. Furthermore, we propose a methodology for replacing sensor data knowledge with artificial intelligence for plant health estimation. Plant health estimation allows longer ebb periods and increases the nutrient level in the final product. With intelligent design and the use of artificial intelligence algorithms, we will reduce the cost of plant research and increase the usability and reliability of research data. Thus, our newly developed greenhouse would be more suitable for plant growth research and production.

## 1. Introduction

Advances in computing technologies based on embedded systems with the recent development in smart sensors are leading to cost-effective solutions for the Internet of Things (IoT). The Internet of Things is an essential component of smart home systems, smart transportation, healthcare, and smart agronomy. In any production environment, especially in agronomy, Internet of Things devices enable efficient planning and resource allocation, providing economic benefits and increasing competitiveness in the market [1,2].

The continuous fusion of computing and agronomy science opened a new field called precision agriculture, leading to higher crop yield within the greenhouse facility [3]. An innovative approach using IoT as a data source and deep learning as a decision maker can optimize the greenhouse environment such as temperature, humidity and nutrients [4]. By monitoring the growing process in the greenhouse, better quality of food, cosmetic products and medicinal substances can be achieved by increasing the plant nutrient levels [5].

According to related work in greenhouse design, the sensors and their location inside the greenhouse are essential components since some parts of the greenhouse contain microclimate pockets. The sensors are organized in several combinations of horizontal, vertical and hybrid arrangements to detect and eliminate microclimate pockets [6]. Additionally, camera positioning system should be flexible enough to allow precise and diverse image acquisition for successful deep learning model training. Image quality, especially noise levels, can reduce the deep learning model precision [7].

In this paper, we present the system architecture and design of a modern scientific greenhouse research facility for the purpose of Croatian Science Foundation’s Project Urtica—BioFuture. Several sensor nodes are proposed in different locations: nutrient solution, environmental (inside and outside the greenhouse), and sensor nodes for energy efficiency and power supply. They are connected via a dedicated central node. As a main contribution, we propose a novel system architecture concept for automated sensor positioning using suspended platform concept to measure accurate environmental data in any available position to achieve the best possible automated hybrid arrangement for microclimate pocket isolation. Using this measurement microclimate pockets will be detected and isolated. The proposed positioning system enables precise image acquisition from multiple angles, thus resulting in image data diversity. Image diversity plays an important role in deep learning model regularization.

Sensor sensing techniques and communication technologies are also considered in this paper. Precise sampling techniques are used resulting in Big Data due to the scientific nature of this data acquisition. To enable a steady flow of this data, a constant power supply and uninterrupted connection are essential. The data is stored locally and continuously synchronized with the cloud service.

The cloud will provide plant health calculations according to sensor data analysis. Opposite to calculation, we propose a methodology to use a deep learning method that uses RGB camera images, chlorophyll leaf images, and thermal camera images to estimate plant health. Such methodology can lead to equivalently precise, yet more affordable solutions applicable in the production. Common benefits of deep learning models and Big Data mining are proactive alerting and monitoring systems or autonomous decision making, which are particularly useful in smart agriculture [8]. Additionally, combining visual data such as images and using sensors to train the corresponding deep neural network model based on visual information proves essential for building an affordable smart agriculture system [9]. Visual information analysis reduces the monitoring complexity and overall price while maintaining the precision achieved with the sensor cluster.

This calculation of plant health is used in the project to optimize ebb timing periods. The decision when to make a phase change is a key issue in the project. The goal is to achieve extended ebb periods for higher plant nutrient levels while avoiding plant wilting. This is the main challenge to be addressed in the upcoming project experiment.

We wrote this paper as part of Croatian Science Foundation’s Project Urtica— BioFuture [10]. The project focuses on the development of a modern greenhouse research facility as a quality basis for future research at the Faculty of Agricultural Sciences, University of Zagreb, Croatia, with the support in computer sciences from Zagreb University of Applied Sciences. This project focuses on the nutritional and functional Urtica Dioica (common nettle) values in modern hydroponic cultivation techniques [10].

This paper is organized as follows. Related work on existing greenhouse solutions is discussed in Section 2. Then, key highlights of the system architecture and design are presented in Section 3. In this section, a detailed description of the sensors and data acquisition follows, highlighting the greenhouse layout where a new sensor data positioning is proposed to capture all microclimate pockets. Later, a cloud communication and storage is described. Finally, the cost of the system is approximated. In Section 4 we presented an experiment with a model of suspended platform. The paper is concluded in Section 5, where the advantages of our proposed system and suspended platform are discussed, and finally some future research directions are given.

## 2. Related Work

The sensor system is a crucial element of smart agriculture. In greenhouse cultivation, especially in the laboratory environment, any value in an experiment can be significant. Majority of the current greenhouse solutions use sensors in different stages of farming for information gathering, effective monitoring and decision making. The main drawback of these greenhouse solutions is the lack of sensory diversification.

Wei et al. [11] presented a review of the current development of technologies and methods in aquaponics. In the greenhouse environment, water quality, environmental data and nutrient information are involved in intelligent monitoring and control. The paper summarizes intelligent, intensive, accurate and efficient aquaponics concepts that we used as a start point for our greenhouse development.

### 2.1. Sensors

In modern scientific greenhouse research experiments, a vast number of different sensors must be used to reduce the possibility of inadequate research results. The significant number of sensors is used to reduce the influence factors on different greenhouse locations and to detect different influence factors in the plant growth. Due to the nature of any scientific development, it is of great importance to keep the expenses within the project limits. Therefore, experimenting with expensive and complicated sensors may be uneconomical in such projects. Additionally, it can be challenging to apply such an environment to production facilities [12]. Various sensors are essential for science-based approaches to smart and precision agriculture. These sensors include environmental, power supply (for energy efficiency), nutrient solution sensors, and sensors that determine the chlorophyll content of plants [13].

Almost all environmental variables (temperature, humidity, amount of light in common and individual spectral regions, atmospheric pressure and air quality) in the greenhouse system can be used as sensed data. Due to the specific requirements of the greenhouse experiment, different types of environmental variables need to be monitored, and thus different values of sensors need to be measured [13]. Many different combinations are sampled based on experience and experimental parameters: Temperature, humidity, CO${}_{2}$ concentration, illumination, illuminance (limited to a specific part of the spectrum). Other sensors include barometric pressure, specific gas concentration (oxygen, nitrogen, ozone) [13,14].

In addition to environmental sensors, there are other sensors that are used to increase the environmental and energy consumption awareness of the project (green-it solutions), resulting in an advantage in economic costs. For this purpose, power supply sensors are used to determine the energy footprint of the greenhouse. The building strategy of the modern greenhouse is focused on equipment, sensors and processes that are energy efficient. Bersani et al. [15] wrote an article on precision and sustainable agriculture approaches that focuses on the current advanced technological solution to monitor, track and control greenhouse systems to make production more sustainable. Pentikousis et al. [16] discusses the communication environment of the sensors to transmit their data and propose server-side data aggregation methods. In addition, the article presents sustainable approaches to achieve near-zero energy consumption while eliminating water and pesticide use.

In production greenhouses, environmental and power supply sensors are used as part of monitoring control processes to delay or accelerate decisions about opening windows, blinds, or switching thermal processes such as cooling or heating. An example of the monitoring and control system is presented in [17,18]. The collected data can be processed using hybrid AI methods [19] or by applying mathematical models [20]. With the usage of the monitoring and control system, a zero-energy footprint can be achieved. In addition, the power supply sensors can be used as an alert medium for a power outage warning, which may cause irreparable damage and loss of scientific research data. As presented in [21], in case of main power failure, adaptive power management can be implemented to extend backup power supply lifespan.

In greenhouses, power supply is used for nutrient delivery to the plants and maintenance of proper level of nutrient solutions in the floating system. Therefore, solution level sensor is used to monitor the level of solution in the floating system [11]. Nutrient solution sensors are used to determine the properties of the nutrient solution. The most common properties measured in the nutrient solution are temperature, dissolved oxygen, total dissolved solids (TDS), and hydrogen strength (pH) values [11].

In the hydroponic floating system, the root of the plant is partially immersed or sprayed in the nutrient solution and in most cases lies in a growing medium. This growing medium draws moisture from the nutrient solution. The moisture content can be measured with a soil hygrometer ( humidity detection sensor) [22] which is inserted into the growing medium. The sensor consists of an EC probe and a soil resistance metric. It is used to measure the electrical resistance of the soil, which is an indicator of soil salinity. The salt concentration of the nutrient solution can change over time, affecting the sensor reading. Therefore, the differential values of the sensor over time are more relevant than directly measured results [23].

A well-balanced plant nutrient growing solution results in a healthier plant. The plant health can be observed by monitoring the visual physiognomy of the plant, and this system can also be used to analyze and detect plant diseases or crop damage [24]. Nutrient solution should be inspected and changed frequently to enhance the elimination of phytopathogens [25].

Visual monitoring ranges from custom-made devices such as LeafSpec [26,27], the use of a normal camera combined with a microcontroller, a processor board [28,29,30] or a smartphone camera [31,32,33]. Papers propose monitoring plants with different types of cameras: standard spectral camera, infrared camera, thermal imaging camera, or color component camera.

A hyperspectral and spectroscopy system camera is used [34,35] to obtain better results. There is also an example of a custom-made system used in [36]. The camera can observe the plant as a whole or just a part of it, such as the leaves. The context is also distinguished by image precision. The image can be taken in a precise position with little background noise, or from a distance with somewhat unpredictable background and viewing conditions.

Opposite to camera systems, RGB color sensors are used in [37,38]. A RGB color sensor or infrared sensor provides a direct numerical value for a specific detail on the captured image.

### 2.2. Data Acquisition

Different greenhouse segments are subject to a specific microclimate pocket, usually caused by the greenhouse orientation, external shading, materials used, materials or other causes. Therefore, sensor positioning and sampling time frames are critical to data acquisition in a modern greenhouse. Specific microclimate pockets affect plant growth and will affect the data if not included in the calculation of the experiment. Therefore, sensors must provide normalized data and microclimate data specific to the position in the greenhouse. Normalized data is collected by using specific models that estimate or interpolate sensor data across the greenhouse [39,40].

Kochhar et al. [6] classified fixed sensor positioning as horizontal, vertical, and hybrid. This type of positioning is not sufficient to capture all microclimate data [41]. Wu et al. [41] proposed a sensor placement model to maximize target coverage without occlusion. As an alternative to fixed positioning, multiple papers propose mobile sensor placement in greenhouses [13,42,43,44,45].

When using an autonomous sensor carrier vehicle, significant attention must be paid to layout optimization for rapid and safe navigation [45]. In paper [46], an obstacle detection system using Kinect sensor is proposed. These sensors are connected to a robotic vehicle that drives around the greenhouse [43]. On a robotic vehicle, an arm can be placed for further reach [42].

In the previous papers, sensors are moved through the greenhouse to detect and measure microclimate pockets. In contrast to the movement of sensors, the plant delivery system is proposed to eliminate the influence of microclimate on plant growth [44]. This complex solution still leaves the influence of microclimate on sensor data. Other works propose the use of drones, especially in plant fields [13].

When using a variable sensor layout, a large amount of data is collected and processed locally or sent to the cloud. This data can be very complex to analyze due to the added component of its locality of acquisition. Data reduction can be achieved by removing repetitive results using sensor data sampling techniques. Similar measurements of the neighboring locality can be excluded if the difference is below the context-specific threshold, which depends on the required data quality. Another approach in the sampling procedure assumes a small hysteresis around the last measurement result. If the result remains within the given frame, it is discarded since no change is detected [47]. There is also a proposal that small anomalies can be discarded [6]. By using the algorithm proposed by Kochhar et al. the sensor frequency sampling can be maximized to capture specific events and redundant data is discarded [6].

Data acquisition, processing, and sampling require computational power in the form of data processing and storage. Computing power board equipped with microcontroller or processor with an operating system is essential to link sensor data and the database. The database can be available on-site or through a connection to a remote database in the cloud. Depending on the requirements, each system can be based on microcontrollers, a processor board with an operating system, or a hybrid system.

Microcontrollers provide better connection interface options with sensors. Most of them are equipped with multiple connection interfaces such as I2C, SPI or UART. The most commonly used microcontrollers are based on Arduino. The most popular Arduino compatible boards include Arduino UNO, Arduino Yun, Arduino Nano, Arduino Mega, ESP8266, ESP32, Intel Galileo Gen 2, Intel Edison, Beagle Bone Black and Electric Imp 003 [48].

Microcontrollers provide direct analogue input interfaces as they are equipped with analogue-to-digital converters. However, they lack storage, multithreading and multiprocessing capabilities. Rabadiya et al. [49] proposed a system implemented using ESP8266 and Arduino support. There are also multiple papers using Arduino boards for data processing in greenhouses [13,50,51].

Another approach opposite to microcontrollers is the processor boards with the operating system. The most common operating systems are specific Linux distributions without graphical interface. In such environments there is the possibility of local database storage with multi-thread and multiprocessing capabilities. The most popular processor boards that include the operating system are Raspberry Pi, Orange Pi, Banana Pi, Odroid. However, these boards have a smaller number of pins than microcontroller boards. They have I2C, SPI, and UART interfaces, but they lack analogue input pins that are equipped with analogue to-digital converters. These types of boards usually have larger power requirements and dimensions. There are hybrid solutions based on a microcontroller board with a tiny OS (e.g., RTOS, MicroPython) [23].

In multiple papers, a combination of microcontrollers and processor boards is proposed to reduce power requirements and provide multiple analogue interface sensors. Systems with lower power requirements are usually based on solar or battery powered concepts [52].

In a combination system, a node consists of a set of microcontrollers that provide sensor interfaces to processor boards that aggregate and send data to the cloud. Each node collects data from multiple sensors connected via interfaces. The nodes can be connected to power, battery or be solar powered. In a combined system, a central node based on the processor board node is required [52].

There is a need for interconnections between the nodes to enable communication. These connections can be classified into wireless and wired. There are multiple wireless standards available for IoT devices. The proposed wireless connection depends on the availability of the microcontroller or processor board interface, the required power requirement, the required connection bandwidth, the communication distance, and the common obstacles in the communication [53]. The connection protocols vary from Bluetooth and WiFi to GSM, radio (NRF) or ZigBee [6,54].

There are also mobile network protocols such as GPRS, 3G, 4G and 5G [55]. A particular protocol can be invented, but it is not a standard solution for use due to incompatibility with other systems. When wireless communication is used, more power node consumption is expected.

In contrast, a wired connection may use a connecting wire to supply power. The most known protocol is power over ethernet. However, there are other options that are not standardized. The wired connection provides an uninterruptible power supply (UPS), which ensures system availability in the event of a power failure. The UPS also provides information about a power failure or low UPS battery to the nodes. This information can be used to gracefully shut down all nodes and alert maintenance personnel in a timely manner.

Each communication is composed of a physical link layer and a logical link layer. The physical link layer can be used as a wired or wireless link. Above the physical layer is a logical layer in the form of a communication protocol. In most simple solutions, a specific protocol can be programmed specifically for the solution at hand. In most cases, standard networking protocols and addressing are used, such as Internet Protocol (IP). IoT devices have standardized specific protocols. The most commonly used specific protocol is Message Queuing Telemetry Transport (MQTT) [50]. Despite the specific IoT protocols, standard web service communication protocols such as HTTP, HTTPS, and SOAP are common.

When working with publicly available services, it is necessary to pay attention to security. In any network architecture, there is a risk of cybersecurity threats. To make a system more secure, Astillo et al. [56] proposed a lightweight specification-based distributed detection to efficiently and effectively identify the misbehavior of heterogeneous embedded IoT nodes in a closed-loop smart greenhouse agriculture system.

### 2.3. Big Data Collection and Deep Learning

The data received from the greenhouse sensor system is stored in the cloud. The cloud allows data to be displayed in time frames and complex analysis to predict greenhouse behavior. The collected data stored in the cloud can be processed by different algorithms in the complex model or fed as training data for a neural network [3]. Kocian et al. [57] predict plant growth in greenhouses using Bayesian network model. Plant growth can be predicted using simple algorithms such as linear regression [58]. Ready decisions or inferences can be used as triggers in other systems, such as smart home implementations as described by Chen et al. [59].

Complex deep neural networks are becoming an indispensable tool for Big Data analysis in a variety of scientific fields, including smart agriculture [60,61,62]. Harnessing the vast amount of data collected over a long period of time enables the training of complex deep neural models. Deep neural network models are one of the crucial approaches used in computer vision. A deep neural model with many parameters can be used for crop classification, yield prediction, and early detection of stress and disease. A considerable amount of computer vision-based work in smart agriculture focuses on plant stress detection, either as disease early detection [63] or water stress detection [64,65,66,67,68].

Plant classification is another important research direction, as it enables the detection and elimination of weeds [69], leading to fully automated cropping systems. Fruit counting [70,71,72] using deep neural networks and computer vision significantly improves yield prediction and automated harvesting. Object detection can be used to detect obstacles in greenhouses, leading to autonomous vehicle passage.

Deep Learning improves weather prediction [73,74], a key to successfully predict weather hazards (storms or floods) that can cause severe damage to the greenhouse. Plant feature recognition as part of plant phenotyping [75] has recently benefited from deep learning models that replace manual work, improving efficiency and effectiveness in precision architecture.

In modern greenhouse research, image analysis using computer vision drastically reduces the need for various sensors and even enables low-cost solutions with few to multiple image acquisition instances [34,35]. Deep learning can assist in clorophile fluorescence estimation, as presented in [76]. To successfully train a deep neural network model, a reliable verification model is crucial. A carefully designed sensor layout is necessary for the successful training and validation of the computer vision neural model. Specific sensors can be used to provide numerical data in correlation with the obtained images [37,38].

## 3. System Design and Architecture

**Figure 1:**
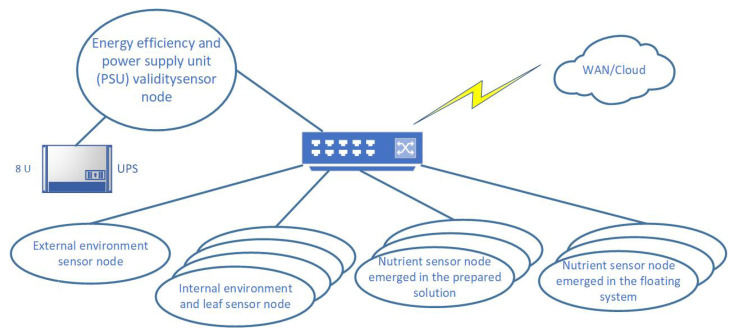
System design and physical architecture scheme. The image describes the organization of major greenhouse nodes with short descriptions. All nodes are interconnected through the local area network and communicate with cloud via the wide area network.

The system design and architecture is presented in the Figure 1. The figure describes the overall architecture of the proposed greenhouse system, and as such it is discussed in subsections throughout this chapter. The design and architecture are described in detail in this section as follows. First, the sensor acquisition is described, then the sensor placement is proposed and discussed. Data acquisition methodology is presented in the third part of this chapter, and finally data acquisition and data storage are presented and described.

### 3.1. Sensor Selection

According to related work, there are a variety of sensors for greenhouse monitoring in agronomy. There are sensors that directly provide data describing the condition of the plants or the nutrient solution state. Values in greenhouse cultivation such as temperature, humidity, light in common and single spectral ranges, air pressure, air quality, soil moisture, soil pH and oxygen saturation can be efficiently monitored with sensors. This wide range of sensors differs in terms of their sensing techniques, electrical characteristics, communication technologies, power requirements, and precision and range. Sensors assembled according to related work can be classified according to their localization in measurement:

- Energy efficiency and power supply unit (PSU) validity sensor node
- External environment sensor node
- Internal environment and leaf sensor node
- Nutrient sensor node emerged in the prepared solution
- Nutrient sensor node emerged in the floating system

The energy efficiency sensor node is based on monitoring the power supply unit. The monitored values are voltage level, current level, power factor, power output and power consumption. We propose to use the digital power meter for measuring voltage, current, power and power values in real time. The power values can be used to estimate the maximum power during the day which is defined as voltage and current in the given time. The power consumption is calculated in a desired time frame and defines the energy required during the selected time period. These two values can define optimal parameters for alternative energy sources. In addition, it allows us to monitor all specific processes in the greenhouse to make them more energy efficient. For energy measurement we propose PZEM-004T electric power meter [77] sensor connected to a smart device via serial interface.

We propose the classification of power consumption in the greenhouse into monitoring, heating/cooling and cultivation processes. The monitoring process allows us to monitor plant growth using several different sensors and processes. The measurements obtained from these sensors provide the information that leads to a decision on the parameters of the nutrient solution and serve as input for other greenhouse processes. The energy requirements of this system depend on the number of sensors, their location, sampling rates, and the technologies used to collect data. The energy consumption monitoring system is essential for the research phase, while in the production environment the greenhouse should have a predictable energy footprint.

The heating-cooling process allows for constant temperature and humidity parameters within the greenhouse. This process is very energy consuming and plays a significant role in plant growth. In the laboratory environment, the maximum allowable temperature and humidity deviations can range from a minimum to no deviation limit.

The cultivation process consists of nutrient solution preparation, water level estimation processes, and transfer of nutrients from storage to a floating system. In this process, the monitoring of the power supply unit is mainly focused on the error message, because the power consumption should not fluctuate significantly. Power failures should be detected to minimize the interruption of nutrient solution levels in floating systems.

External environmental sensor nodes outside the laboratory greenhouse measure meteorological data. This data is used to estimate the energy efficiency of the greenhouse by comparing the energy consumption for heating or cooling the greenhouse to the desired temperature and humidity. This node additionally provides readings on the intensity of the light spectrum and the general air quality. The sensor node consists of CO${}_{2}$, temperature, humidity, pressure, multichannel gas sensor, ultra-violet (UV) and visible light, and sensor for visible light with IR cut filter. Sensor selection, measurement range and accuracy were estimated from previous data measured manually in the greenhouse.

The internal ambient and leaf sensor node is mounted above the floating system. The collected data is used to control the internal greenhouse processes. Internal greenhouse processes are heating, cooling, opening windows, ventilating and blocking out external light. They are used to set the preferred temperature, humidity, CO${}_{2}$ level and light intensity in the IR, visible and UV spectral range. This sensor node consists of similar set of sensors similar to external sensor node, additionally equipped with RGB and thermal camera, and RGB color sensor.

The measured data are used to assess the plant environment and thus influence plant health. Due to the microclimate behavior of the greenhouse, the position for the internal sensor node should be accurately determined according to related work. The internal sensor node is equipped with a leaf sensor node, which contains a thermal imaging camera and a visible camera without IR-blocking filter. The camera images are used to detect the chlorophyll and nutrient content in the leaf expressed in numerical values. The position of the camera sensor is crucial to provide higher quality images without noise. The internal sensor node must be positioned over the plant or next to the growing plant to produce images from different angles.

The sensor node is equipped with an RGB color sensor to accurately detect the color of the leaf when it is illuminated from above, according to the related work [37,38]. The obtained sensor data is used as training data to build an AI model that estimates the data from images only. In the later stage, the sensor data is used to verify the model predictions.

Nutrient sensor nodes created in the prepared solution and nutrient sensor nodes created in a floating system provide information about the state of the nutrient solution. Hydroponic system sensors include temperature, levels of PH, dissolved oxygen, total dissolved solids (TDS) sensor, and moisture sensor inserted into the growing media. A level sensor is used to monitor and alarm about the level of nutrient solution in the floating system. A laser range sensor is used to accurately monitor the level of the nutrient solution in a low light environment.

### 3.2. Sensor Placement

Sensor placement represents how the sensors are arranged in the greenhouse. In the literature, sensor placement is often referred to as layout or greenhouse layout. Placement focuses on the physical location of the sensors rather than the topology of the system, which describes the flow of information between sensors, microcomputers, and the cloud.

Sensor placement is a major factor that needs to be implemented carefully, as described in related work. The inside of a greenhouse is a dynamic environment where temperature differences during the plant growth cycle or air flow adjustments can affect the outcome of the sensors. A large greenhouse may have several microclimate pockets that may vary in location or intensity over periods of time.

According to related work, there is a well-known conventional horizontal and vertical sensor positioning system [6]. Besides horizontal and vertical positioning, there are also hybrid solutions such as shelves, boxes, tier-based and master-slave solutions. These solutions try to eliminate the microclimate effect by excluding it from the experiment (plants near the greenhouse walls are not included in the measurement results) or by measuring the microclimate effect in each position [13,42,43,44,45].

An automated robotic vehicle equipped with environmental sensors is proposed to provide data in different parts of the greenhouse [45]. The advantage of this solution is a horizontal coverage of the greenhouse. The disadvantage is a measurement of a certain vertical plane near the greenhouse floor. In case of table experiments, vehicle sensor plane and camera angle may become useless. Even with dynamic vertical positioning, vertical and horizontal positioning is limited due to the inaccessible hover system and plant growth areas. This approach is also not feasible in greenhouses without level ground, as the vehicle can be problematic to navigate.

Other approaches propose the use of a drone (rotorcraft) that can be flown autonomously or manually [13]. Integrating sensors into an unmanned drone system can introduce multiple sources of bias and uncertainty if not properly accounted for [78]. For example, a measurement may be incorrect due to drone thrust, temperature, humidity, and gas levels. Measurements can be mathematically adjusted in a laboratory setting with additional experiments. The drone system poses an additional safety risk, as people or plants in the greenhouse could potentially be damaged during flight. If continuous sampling is required, drones (especially heavily equipped ones) consume a lot of energy, so flight time and battery charging time can become an issue.

To mitigate shortcomings of the classic horizontal and vertical sensor placement, different automated robotic vehicle concepts, or even sensor equipped drone techniques, we propose a solution to implement a suspended platform with the sensor node. With this approach, we eliminate the problem of uneven greenhouse ground or other obstacles which can appear on greenhouse floor such as water piping or other infrastructural objects. Moreover, with constant power supply, battery duration is not an issue, compared to autonomous vehicle or drones. Side effect of positioning is minimal opposite to drones which generate air turbulence and affect the measurements. The concept of suspended platform is inspired by the mechanical design of a CNC machine table or a 3D printer. This design is very rigid, and it may affect the sunlight of the plant by blocking it. It is more difficult to assemble due to the lightweight construction rods of the greenhouse.

**Figure 2:**
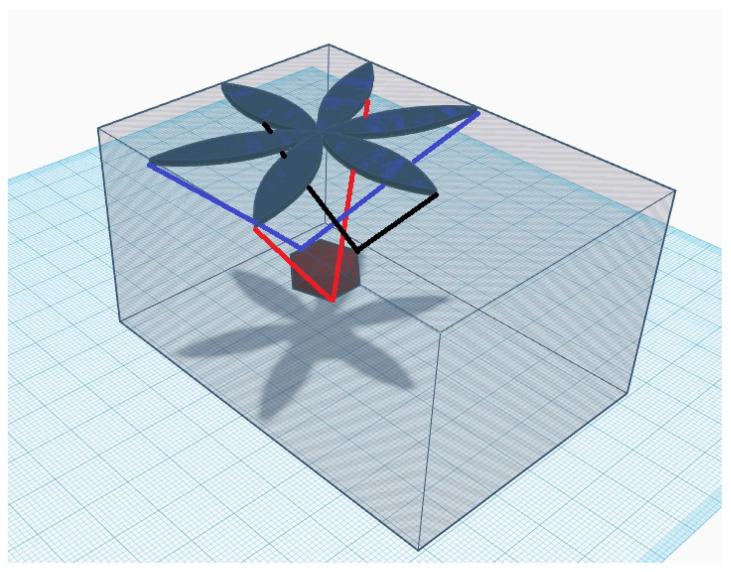
The proposed suspended platform concept. The suspended platform uses a six-degree-of-freedom cable-suspended robot for positioning. Cable-positioning systems can be easily applied in different greenhouse layouts since they provide large ranges of motion.

To solve these problems, a new concept of a hanging 3D positioning system is proposed based on a novel approach to large-scale 3D printing [79]. This concept allows the suspended platform to be suspended with sensor nodes and controlled by attached wires. To enable 3D oriented positioning, wires are attached from the suspended platform to the ceiling and diagonally to the angles of the greenhouse. The system is shown in Figure 2.

Suspended sensor node allows the sensor node to be placed in any possible position above the floating system by manipulating the X (width), Y (length), or Z (height) coordinate. The experimental system can be programmed to automatically position the sensor node over horizontal and vertical positions to obtain results from different microclimate pockets. Using the results data in time intervals, specific microclimate pockets can be identified, and their variations estimated.

Internal environmental and leaf sensor nodes in the laboratory greenhouse are placed together on a suspended platform. The suspended platform is used to detect microclimate pockets as their position changes, and cameras simultaneously capture images of the plants. Using an automatic guidance system for the suspended platform, plant images are captured in time frames and uploaded to the cloud. At the same time, the real data is measured and linked to the images in a database. This technique can be used for data preparation for the AI learning process and later as a verification technique. Additionally, microclimate pockets can be discovered by analyzing this data.

The nodes of the external environmental sensors outside the greenhouse should be placed in an optimal position, e.g., above the roof or in a more remote location without the influence of internal factors of the greenhouse. In our case, one external environmental node is sufficient because the greenhouse is directly exposed to the sun without any obstacles. If the greenhouse has a specific orientation or obstacles that partially block part of the greenhouse during the day, multiple sensor nodes would be a mandatory solution.

The nutrient sensor node that has emerged in the prepared solution is placed on the floating platform inside the holding tank. Nutrient sensor nodes that have emerged in the nutrient solution for plant growth are placed on the floating platform within the floating system. Nutrient sensors require special treatment due to sediment formation on the probes. pH and oxygen probes should not be continuously immersed in the nutrient solution. After successful measurement, the probes must be removed from the nutrient solution and immersed in clean distilled water before used in the same or a different nutrient solution. The cleaning process of the probes can be done manually or automatically using the robotic arm concept. We propose using high-quality probes that can be immersed in the nutrient solution for extended periods of time without negatively affecting the measurement results.

### 3.3. Data Sampling

The data sampling procedure is used in plant analysis, where a predetermined number of data points are taken from a more comprehensive set of observations. The sampling procedure is very specific to the type of sensor and its interface. To properly document changes in the parameters sampled, sampling should be done at optimal time intervals. The limitation of the sampling frequency is determined by the interface type or the specific sensor technology.

The interface type determines the connection speed, but this is limited by the sensor technology or the common bus throughput when multiple sensors are connected. For example, the direct digital interface, analogue-to-digital converter, serial interface, I2C, and SPI interface have different data flow speed limitations. For multiple devices, the speed is divided by several devices on a common bus. The datasheet is analyzed for each sensor and interface, and the maximum sampling speed is presented in Table 1. Additionally, the average sensor cost is presented in table. There is an additional time limit for the first measurement in the case of a pH or dissolved oxygen sensor. These limitations are presented in Table 1.

**Figure 3:**
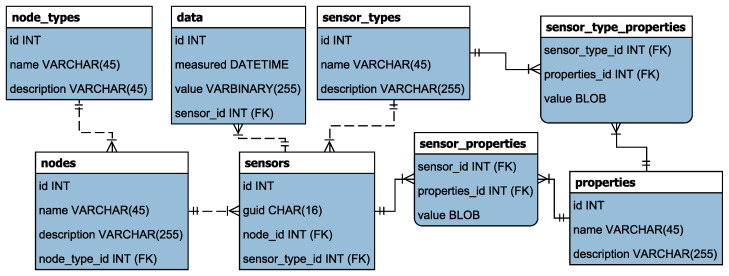
ER model of the local sensor node database.

Each sensor node has its own computing power for data analysis and local data storage. Proposed computing power is a Raspberry Pi with MySQL database equipped with additional scripts. The scripts enable interaction between the sensor interface and the database. They are also responsible for the communication between the local storage and the cloud [52]. The local sensor node database defines the sampling interval, the location of the script, the location of the local database, the deviation range, and additional sensor data, which are presented in Figure 3. The system is run from a central execution script written in Python that runs multiple scripts for each available sensor.

An exception to storing in the database are images which are stored in the local file system. Images are not stored in the database because the database engine is not capable of handling a large blob. Path and name are placed in the local database table instead of result data to track images stored in the file system.

Script queries sensors and stores result in local database along with current timestamp. Nodes are synchronized with atomic clock daily to ensure accurate timestamp. All scripts are adjusted to discard values that deviate significantly from the estimated threshold during test measurement periods. Repetitive values are not recorded because they take up space in the database and would slow down query execution. Their absence from the database does not affect the final result, as the system assumes that the value has not changed during the queried period.

Smaller deviations can be caused by sensor fluctuation, which is common with analogue-to-digital converters due to the specific measurement process. Fluctuation can also be caused by sensor-specific measurement techniques or properties of the media being sensed, such as sensor purity, water movement, air flow, or light reflection. These fluctuations do not need to be stored in the database as they have no direct influence on the plant growth process. The fluctuation limit must be carefully estimated from the sensor data sheet and the empirical measurement process.

A high deviation means that an alarm must be triggered for sensor inspection. These deviations can be caused by contamination of the sensor, movement (out of the medium or out of range of the sun) or technical malfunctions. Reported alarms are automatically processed in the cloud and forwarded to maintenance. Due to the potentially significant impact on the plant growth process, a quick response is required in some cases (nutrient solution level or temperature).

The sensor nodes need to communicate efficiently with the cloud. This process introduces a compression algorithm with or without data loss to reduce the data flow to the central database. For a large amount of data, a NoSQL database [94] is recommended. In our greenhouse model, a SQL database is used as a local buffer to provide accurate alerting due to limited storage capacity. The cloud database will be based on NoSQL due to the large amount of data. A warehouse model of the collected data can additionally be built for specific time periods.

### 3.4. Data Collection

Each sensor node has local database storage, file system storage, and processing computing power. All nodes are connected to the wired Internet. The wired Internet is used to ensure continuous connectivity, as a wireless connection has a higher interference rate. A wired interconnect cable is used to provide power through a method known as power over ethernet. This method uses four wires that are not used in a standard 100 Mbps ethernet connection. The non-standard power supply voltage is used (12 V) to power the computing node, sensors, and motor system of the suspended platform. The available voltage (12 V) is rectified within the node into other required voltages according to the data sheet of the sensors. In this way, connectivity and power are provided simultaneously through a cable connection with a central power supply. The proposed sensors have low power requirements and do not require a high current throughput cable (large cross section). The use of batteries or solar cells is not practical, even in combination with a microcontroller and sensor sleeping functions, since the motors of the suspended platform require a significant amount of energy to wind the cables.

A sensor node is provided as a central node. Based on the position of the nodes, the central position node is the energy efficiency and power supply node. This node is closest to the wired wide area link and power supply and has an additional sensor to check the availability of the main power supply. The entire system is connected to the main cable via the uninterruptible power supply (UPS), which has a serial interface to communicate with the energy efficiency and power supply sensor node. In the event of a main power supply failure, the system operates without interruption for a certain time frame defined according to the UPS capacity. The UPS uses its battery power instead of the main power supply and sends information to the energy efficiency and power supply node in case of a power failure. When a power failure is detected, the energy efficiency and power supply node alerts the maintenance staff to verify the reason for the power failure. In our case study, the proposed time frame is eight hours to enable timely maintenance response.

The UPS informs the energy efficiency and power supply node to start shutdown requests that propagates to other sensor nodes as soon as the battery power decreases. Since all nodes are equipped with the operating system, local database, local memory, and scripts on the SD board, a graceful shutdown is expected. In the event of an immediate power failure, there is a possibility that the file system will be corrupted and thus the operating system will not boot. Each node acknowledges the orderly shutdown request and starts the shutdown process. After losing network connectivity with the sensor node, the energy efficiency and power node knows that a graceful shutdown has been completed on a sensor node. After determining that all nodes have completed the shutdown process, the energy efficiency and power supply node will shut down. This process must begin in time before the complete power failure of UPS to complete successfully. The shutdown period must be extended as the batteries of UPS lose capacity over time.

Energy efficiency and power nodes inform maintenance personnel with alerts of the following priorities: fatal, technical, and anomaly. Fatal faults such as power supply failure are immediately sent to maintenance personnel. Technical and anomaly faults are collected and presented to cloud users upon connecting. Technical faults are associated with technical system architecture and maintenance. Anomaly faults are linked to outlier sensor readings. Low priority errors may increase. For example, an incorrect sensor reading is an anomaly fault. If multiple anomaly warnings are repeatedly detected within a short period of time, an anomaly fault is elevated to a technical fault. If an anomaly is detected over an extended period of time, it is upgraded to a major fault because it may indicate equipment failure and require intervention.

All sensor data is collected and stored in the local database for each sensor node. The energy efficiency and power supply node hold information about other sensor nodes and local sensor data. This data needs to be transferred to the cloud for detailed analysis.

### 3.5. Cloud Data Storage and Analysis

Cloud-based data storage is an obvious requirement for any potentially distributed system configured to collect data in short time intervals. This is especially true for images, where on-site storage can quickly become insufficient, limiting scalability. Today, the price of cloud storage makes such data storage affordable for almost any budget, guaranteeing data availability and the necessary infrastructure support for low-latency data access.

**Figure 4:**
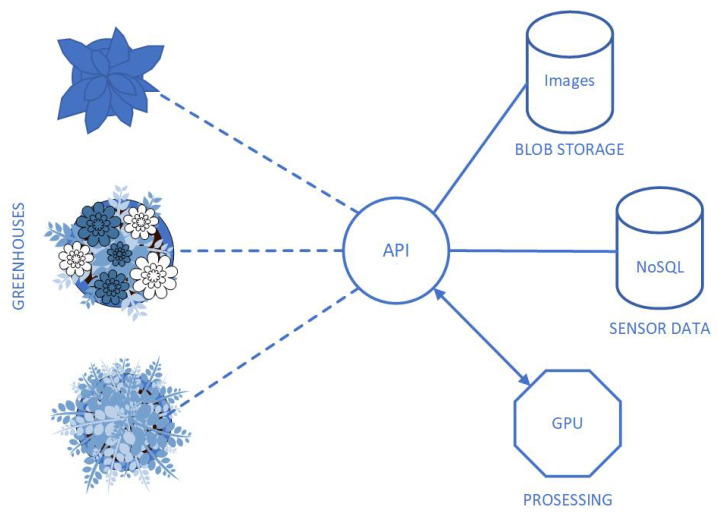
The high-level system architecture.

The cloud receives the data through a publicly accessible web service point protected by a standard authentication mechanism and a whitelist for IP addresses. Data is transmitted as simple JSON and stored in the NoSQL data store due to direct compatibility with JSON format. Images are uploaded in RAW format, which is referenced in the JSON data and stored in the cloud blob storage. The local sensor nodes organize the data and upload it to the cloud immediately using the sampling process. In case of possible network failure or server problems, the data is stored locally for a longer period of time to avoid data loss. The proper period for local storage is empirically estimated and depends on the sampling process and hard disc capacity. The received data is analyzed in the cloud to determine the state of the system. The high-level system architecture is presented in Figure 4.

The data obtained from the greenhouse is organized and summarized to analyze the dependent and independent variables of the process. The deep neural network model acts as a high order nonlinear function that determines plant health based only on simple camera images. This may include a deep neural network model based on a thermal image and multiple dependent basic color (RGB) images of the camera without infrared filters. In addition, a data warehouse solution is available to support the need for recurring data reports for specific time periods.

Plant health will decide when the end of the ebb period is reached, as plant health deteriorates with prolonged ebb periods. The decision made in this way should extend the ebb periods as much as possible and thus, according to previous research in agronomy, provide a plant with better nutritional values [95]. There will be other floating systems with fixed ebb periods that will act as an experimental control group during this experiment. Plant health will also be calculated for them.

Plant health will be determined in two different processes. The first process involves a deep neural network model that estimates plant health by analyzing greenhouse images. The second process estimates plant health mathematically based on sensor readings provided by the greenhouse. The data obtained from the second process is used as a correction factor for training and fitting the deep learning model. Calculating plant health only from multiple statically placed camera devices significantly reduces the implementation cost of the greenhouse.

Even without the sensor node system, it is possible to detect a malfunction of the system based on the calculation of plant health over a period of time. During this period, sudden deviations in the plant health calculation will alert the researchers because there is a possible problem with the proposed calculation model or serious problems within the greenhouse system, such as nutrient solution level, temperature, or artificial light error. Ultimately, the images processed with the deep neural network model should be sufficient to replace the sensor node system for determining plant health in the production greenhouse.

### 3.6. Deep Neural Network Model

Deep learning models usually contain a considerable number of trainable parameters that take a long time to train. In the context of computer vision, inference can also be the bottleneck. Although there is significant development in edge computing and optimizing such models to run in the field and even on embedded devices, for optimal results, a high-end computing device should be used to achieve real-time or near real-time inference speed. Even with a fast CPU, deep learning models can take a significant amount of time to evaluate, so GPU computing units that support a high degree of parallelism and are optimized for running complex deep learning models are needed. Large-scale smart farming systems typically do not require real-time processing. Nevertheless, the cloud solution enables cost-effective on-premises sensor and camera equipment and provides the ability to simultaneously support multiple distributed deployments with centralized AI analysis nodes. Once the deep-learning-based model processing is complete, the data is stored and made available for any further data processing. In fact, the system is designed to retrain the model with a larger amount of data when enough new data is collected, increasing the efficiency and precision of the model.

Supervised learning is a simple approach in the given system, mainly due to high availability and a large amount of ground truth data—plant health value—calculated from a reliable sensor source. For image processing, the deep learning model consists of a backbone based on convolutional neural networks using one of the proven backbone architectures such as ResNet [96], Inception [97], DenseNet [98] or an efficient concept of backbone network scaling [99]. Since the plant health value is a single number, the model contains a regression head with MSE loss function. Due to catastrophic forgetting, small periodic model updates are not easy to achieve. Therefore, we tended to use large periodic updates over a longer period of time. We leave a detailed analysis of the model update time frame to future work.

### 3.7. Implementation Cost Analysis

For the described smart greenhouse architecture to be competitive in the market, cost estimate should be included. The expenses can be divided into setup expense and operational cost. Setup or installation cost includes the sensor set cost, RGB and thermal camera, Internet connection installation (if missing) and suspended platform mounting. Table 1 shows the estimated cost breakdown per sensor. The sensor cost can be reduced by using the AI module to estimate the sensor values based on RGB plant images. Operational cost includes the Internet connection rates, data storage and compute cost, and GPU processing cost for AI image analysis. Depending on the data retention and level of sampling the storage and compute cost can be somewhat adjusted to specific needs. GPU processing in pay-as-you-go pricing models would require approx. 200–300 ms GPU processing time per image analyzed. Image analysis frequency can also be reduced if measurements follow a predictable pattern or high precision of not of essence. The cost of model training is not included as it is performed once during the research, and henceforth the trained model will be used only for inference.

## 4. Experimental Findings

In every greenhouse the temperature and the humidity are measured. These two sensors form the minimum measurement setup, although each specific greenhouse might require a specific set of sensors. Each sensor from set provides specific values that depend on the element measured. Very often, the elements measured depend on the measurement position and corresponding spatial variations. For instance, the temperature next to a window or door, next to a glass wall or in a corner in the shade will report different results. The measurement differences acquired this way form microclimate pockets.

**Figure 5:**
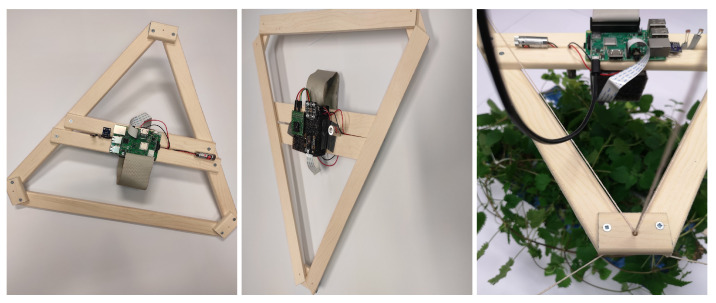
Suspended platform model. View from above and below on mounted internal sensor node. Suspended platform model during experimental positioning—test of cameras and platform stability during image acquire.

Therefore, the sensor positioning within the greenhouse is extremely important, since our primary goal is to locate and isolate the microclimate pockets. Variations measured in the microclimate pockets affect plant health and should be included in the calculations and data analysis. This requires automated sensor positioning as opposed to the horizontal or vertical fixed positioning. Related research focuses on autonomous vehicles, conveyors and drones to find and isolate microclimate pockets. We have proposed the suspended platform architecture that allows flexible spatial positioning, covering all three spatial dimensions as it can be seen in Figure 5. Additionally, the flexible positioning concept is essential to ensure diverse plant image acquisition to improve deep learning model applicability to new and unseen environments.

The suspended platform is equipped with internal sensor node sensors. The sensor node consists of CO${}_{2}$, temperature, humidity, pressure, multichannel gas sensor, ultra-violet (UV) and visible light, sensor for visible light with IR cut filter and RGB color sensor to detect leaf color. Additionally, to collect images, RGB and thermal camera are attached. During installation, sensors are mounted to prevent the influence on the camera’s field of view. Due to the suspended platform positioning concept, sensor node heat output or sunlight blockage is not an issue since positioning in single location is short. This makes our proposed suspended platform very flexible and precise while not being invasive for plant or plant environment.

In previous articles, we found mainly targeted measurements of microclimatic points based on the specific orientation of the greenhouse or some specific parts such as curtains, blurred windows, or a more densely placed structure. We suggest another approach to divide the plant growing area into the grid of 50 × 50 cm squared zones. Decreasing the square size, positioning system requires frequent calibrations and the time to visit the entire grid increases and becomes non-viable, especially for larger greenhouses. For certain types of sensors, the measurement itself does not occur momentarily, but a certain amount of time must pass before the value stabilizes (e.g., temperature). Overly granulated grid can lead to inaccurate data because the measurement times for different squares are not visited often enough. The size of the grid square should not be too large, otherwise microclimatic pockets might not be precisely isolated.

For a proposed square size of 50 × 50 cm, we can conclude that the allowable deviation of the suspended platform positioning is equal to half the side of the square: 25 cm. The suspended platform is implemented as a cable-driven parallel robot. Essentially, it is a set of at least 6 cables that are wound and unwound by winches and connect a frame and a platform. By synchronously adjusting the length of the various cables, the load can be moved smoothly over a wide area of the footprint, with control and stability in all 6 degrees of freedom.

To confirm the concept and determine the variations in positioning, we propose an experiment to build a model of the suspended platform and to test its positioning abilities. Three laser pointers are mounted on the platform and the printer is guided through wires by hand to specific position. Each laser pointer covers one axis: the display on the right wall, the display on the end wall, and the display on the ground. Each time the suspended platform was moved, we marked the previous point and measured the deviation of the new position from the previous point. Through several cycles of guiding, we reduced the results to acceptable average of 2.7% ± 2% deviation in positioning after full grid positioning cycle and before next calibration. The measurement provided allows for grid size slightly over 800 cm between opposite grid sectors. Additionally, in our laboratory surroundings we tested the possibility of positioning in diverse location, especially near the corners of the laboratory. Precise height positioning of the suspended platform is also satisfactory to be able to provide a closer leaf inspection. With the model experiment we established that it is possible to cover the laboratory ground except for corners.

As a comparison, positioning deviation for the similar process in the field of 3D printing spans up to 1.5%, with isolated outlier of 9.4% [79]. We believe that after motorization with additional calibration, through test experience the better results can be achieved.

The network cable provides power and secures the network connection to the internal sensor node located on the platform. Although we have designed the cable to be flexible, it is obvious during positioning that it affects the balance of the suspended platform, since the results are slightly improved in the experiment without the ethernet cable. After a few tests we abandoned the use of ethernet cable and decided to use a wireless connection instead. In the presented figure only power cable provided as connection to suspended platform.

Our experimental testing on model also showed that the flax cable is more suitable than thin rope as a wire guide. There is a possibility to power the internal sensor node through two wires from which the platform is suspended. This solution would be tested later by replacing two wires with thin steel cable.

This device to be able to reset its positioning should have a zero point. The edge zero point is extremely impractical for the zero point. For this reason, we propose to install a 3-axis sensor and secure a point in the greenhouse where the unique position of the suspended platform can be confirmed. The sensor could be implemented via ultrasound or laser. In addition, such a sensor could detect obstacles during the movement of the platform and stop the operation of the device, i.e., avoid the obstacle by positioning it via the second axis. With this experimental finding we can conclude that suspending platform can be used to detect microclimate pockets and to provide diverse and high-quality close-up plant images for deep learning model training as presented in Figure 5.

## 5. Conclusions

With a higher market for organic food production, there are demands for greenhouse growing in sterile environments, pesticide and fertilizer free, which is hard to find in our surroundings. The integration of IoT devices into non-computational domains provides the opportunity to obtain Big Data analytics of every measurable section of an internal greenhouse process. Such analysis with deep learning models provides valuable insights and scientific knowledge [100].

The main goal of this paper was to present a state-of-the-art scientific greenhouse research facility that can be used during and after Project Urtica-BioFuture. In this paper, we have analyzed related work to gain knowledge about the most commonly used sensors and greenhouse equipping projects in precision agriculture. A detailed sensor node system architecture to cover all internal greenhouse processes and to obtain Big Data, which is subsequently analyzed in the cloud, is presented. The system architecture is presented to describe the design of the components and their interconnection.

The collected data is synchronized with the cloud in real time, which enables additional calculation in the cloud. A deep neural model will be trained on sensor data to estimate plant health from RGB camera images only. This is one of the primary Project Urtica-BioFuture goals. The trained model can be used as a replacement for the sensor system to make the greenhouse system more energy and cost efficient in the production environment.

Microclimatic influences can become a problem in measurement evaluation. To detect microclimates, different layouts for sensor organization are proposed. In this paper, we propose an automated hybrid sensor layout based on a suspended platform to detect microclimate pockets. The proposed layout covers the greenhouse area and allows precise positioning throughout the greenhouse. In addition, it allows camera positioning above the plants thus enabling better plant coverage.

The automated hybrid layout with suspended platform offers the advantage of positioning the sensor node above the plant growing area in all axes. With the introduction of the system, we eliminate problems with fixed horizontal and vertical layouts, problems with expensive conveyor systems, problems with floor leverage and obstacles with automated robotic vehicles, and sensor compensation by drone propulsion. In addition, the proposed suspended platform is powered by wires, eliminating the concept of battery replacement and recharging.

To validate the concept, we conducted a simple experiment by building a model of the suspended platform. In this experiment, we verified positioning errors to confirm the use of the system according to the proposed grid system over the plant growing area in the greenhouse. During the experiment, we also identified raised problems and made suggestions for them. We believe that this paper will enable us to collect better plant images for AI and detect microclimate pockets and enabling their elimination. This would make the proposed greenhouse system more effective and provide a novel starting point for the Urtica-BioFuture project.

For future work, we propose several possible avenues. A detailed analysis of the microclimate pockets in the greenhouse to obtain a mathematical model describing their influence in the surrounding areas of the greenhouse is worth considering. With analyses of the collected data, the sensor system can be further optimized by eliminating or introducing an additional sensor to replace the sensor group. The deep neural network model can be further optimized to provide exact mathematical model for plant health calculations by collecting additional training data from multiple greenhouses and different plant crops. With this approach a sensor data network simplification with the introducing of a deep neural network model will be achieved.

## Figures and Tables

**Table 1:** Used sensors according to related work.

Sensor	Range	Accuracy	Interface	First Measurement	Sampling Speed	Cost
BME280 temp. [80]	−40 °C +85 °C	±0.5 °C	I2C SPI	1 s	1 s	€12.55
BME280 hum. [80]	0% RH 100% RH	±3 RH	I2C SPI	1 s	1 s	€12.55
BME280 pressure [80]	300 hPa 1100 hPa	±1%	I2C SPI	1 s	1 s	€12.55
CO${}_{2}$ NDIR [81]	0 ppm 5000 ppm	±3%	Analog	3 min	120 s	€49.45
UV VEML6075 [82]	Sensitivity: 365 nm, 330 nm	±10 nm	I2C	50 ms	50 ms	€14.55
Light VEML7700 [83]	0 lux 120,000 lux	0.0036 lux	I2C	1100 ms	1100 ms	€4.50
GAS sensor: CO, NO_2_, C_2_H_5_OH, VOC [84]	1 ppm 5000 ppm	Depend on GAS	I2C	30 s	60 s	€40.90
		and concentration				
PZEM004T Energy power meter [77]	80 V–260 V 0 A–100 A 0 W–22 kW	1.0 grade	Modbus-TTL	1 s	1 s	€9.70
	0 Wh–9999 kWh 45 Hz–65 Hz					
PiNoIR camera module v2 [85]	8 MPixel Sony IMX219 NO IR filter		Camera port	30 fps	30 fps	€30.30
FLIR LWIR Micro Thermal camera	80 × 60 resolution	<50 mK sensitivity	Module SPI	30 fps	30 fps	€204.50
module 2.5 [86]						
DS18B20 digital temp. [87]	−10 °C +85 °C	±0.5 °C	I2C	1 s	1 s	€9.70
TDS Sensor [88]	0 ppm 10,000 ppm	±10% F.S.	Analog	1 s	1 s	€10.05
pH Sensor [89]	0 pH 14 pH	±0.1 pH	Analog	1 s	1 s	€84.35
Dissolved Oxygen Sensor [90]	0 mg/L 20 mg/L	±10% F.S.	Analog	1 s	1 s	€144.00
Turbidity Sensor [91]	0 NTU 3000 NTU/L	±10% F.S.	Analog	1 s	1 s	€8.45
Soil Moisture [22]	1.2 V 2.5 V	N/A	Analog	0	0	€5.05
RGB Color Sensor TCS3200 [92]	R G and B values 0–255	±0.2%	Digital TTL	1 s (protocol)	1 s (protocol)	€6.75
Laser sensor [93]	0.012 m 2.16 m	±1 cm	UART	0	0	€21.30

## Data Availability

Not applicable.

## References

[1] Masoud S., Chowdhury B.D.B., Son Y.J., Kubota C., Tronstad R. (2019). Simulation based optimization of resource allocation and facility layout for vegetable grafting operations. Comput. Electron. Agric..

[2] Maksimovic M. (2018). Greening the Future: Green Internet of Things (G-IoT) as a Key Technological Enabler of Sustainable Development. Internet of Things and Big Data Analytics Toward Next-Generation Intelligence. Studies in Big Data.

[3] Somov A., Shadrin D., Fastovets I., Nikitin A., Matveev S., Hrinchuk O. (2018). Pervasive Agriculture: IoT-Enabled Greenhouse for Plant Growth Control. IEEE Pervasive Comput..

[4] Guillen M.A., Llanes A., Imbernon B., Martinez-Espana R., Bueno-Crespo A., Cano J.-C., Cecilia J.M. (2021). Performance evaluation of edge-computing platforms for the prediction of low temperatures in agriculture using deep learning. J. Supercomput..

[5] Ayaz M., Ammad-Uddin M., Sharif Z., Mansour A., Aggoune E.M. (2019). Internet-of-Things (IoT)-Based Smart Agriculture: Toward Making the Fields Talk. IEEE Access.

[6] Kochhar A., Kumar N. (2019). Wireless sensor networks for greenhouses: An end-to-end review. Comput. Electron. Agric..

[7] Kramberger T., Potočnik B. (2020). LSUN-Stanford Car Dataset: Enhancing Large-Scale Car Image Datasets Using Deep Learning for Usage in GAN Training. Appl. Sci..

[8] Ghosh A., Chakraborty D., Law A. (2018). Artificial intelligence in Internet of things. CAAI Trans. Intell. Technol..

[9] Story D., Kacira M. (2015). Design and implementation of a computer vision-guided greenhouse crop diagnostics system. Mach. Vis. Appl..

[10] (2020). URTICA—BioFuture. http://urtica.agr.hr/en/naslovnica-english/.

[11] Wei Y., Li W., An D., Li D., Jiao Y., Wei Q. (2019). Equipment and Intelligent Control System in Aquaponics: A Review. IEEE Access.

[12] Saiz-Rubio V., Rovira-Más F. (2020). From Smart Farming towards Agriculture 5.0: A Review on Crop Data Management. Agronomy.

[13] Miranda J., Ponce P., Molina A., Wright P. (2019). Sensing, smart and sustainable technologies for Agri-Food 4.0. Comput. Ind..

[14] Wei L.Y., Sheng-Kai T., Jyun-Kai L., Ta-Hsien H. (2020). Delopoing Smart Home Applications. Mob. Netw. Appl..

[15] Bersani C., Ouammi A., Sacile R., Zero E. (2020). Model Predictive Control of Smart Greenhouses as the Path towards Near Zero Energy Consumption. Energies.

[16] Oliver P., Kostas B., Calvo R.A., Papavassiliou S. (2010). Mobile Networks and Management.

[17] Wang L., Wang B. (2020). Construction of greenhouse environment temperature adaptive model based on parameter identification. Comput. Electron. Agric..

[18] Subahi A.F., Bouazza K.E. (2020). An Intelligent IoT-Based System Design for Controlling and Monitoring Greenhouse Temperature. IEEE Access.

[19] Castañeda-Miranda A., Castaño V. (2020). Smart frost measurement for anti-disaster intelligent control in greenhouses via embedding IoT and hybrid AI methods. Measurement.

[20] Villarreal-Guerrero F., Pinedo-Alvarez A., Flores-Velázquez J. (2020). Control of greenhouse-air energy and vapor pressure deficit with heating, variable fogging rates and variable vent configurations: Simulated effectiveness under varied outside climates. Comput. Electron. Agric..

[21] Vamvakas P., Tsiropoulou E.E., Vomvas M., Papavassiliou S. Adaptive power management in wireless powered communication networks: A user-centric approach. Proceedings of the 2017 IEEE 38th Sarnoff Symposium.

[22] (2019). DFRobot, Gravity: Analog Capacitive Soil Moisture Sensor-Corrosion Resistant SEN-0193. https://www.dfrobot.com/product-1385.html.

[23] Angelopoulos C.M., Filios G., Nikoletseas S., Raptis T. (2019). Keeping Data at the Edge of Smart Irrigation Networks: A Case Study in Strawberry Greenhouses. Comput. Netw..

[24] Dong Z., Men Y., Liu Z., Li J., Ji J. (2020). Application of chlorophyll fluorescence imaging technique in analysis and detection of chilling injury of tomato seedlings. Comput. Electron. Agric..

[25] Malewski T., Brzezińska B., Belbahri L., Oszako T. (2019). Role of avian vectors in the spread of Phytophthora species in Poland. Eur. J. Plant Pathol..

[26] Wang L., Jin J., Song Z., Wang J., Zhang L., Rehman T.U., Ma D., Carpenter N.R., Tuinstra M.T. (2020). LeafSpec: An accurate and portable hyperspectral corn leaf imager. Comput. Electron. Agric..

[27] Ma D., Wang L., Zhang L., Song Z., Rehman T.U., Jin J. (2020). Stress Distribution Analysis on Hyperspectral Corn Leaf Images for Improved Phenotyping Quality. Sensors.

[28] De Castro A., Madalozzo G.A., Trentin N.S., Costa R.C., Calvete E.O., Spalding L.E.S., Rieder R. (2020). BerryIP embedded: An embedded vision system for strawberry crop. Comput. Electron. Agric..

[29] Yu Z., Ustin S.L., Zhang Z., Liu H., Zhang X., Meng X., Cui Y., Guan H. (2020). Estimation of a New Canopy Structure Parameter for Rice Using Smartphone Photography. Sensors.

[30] Ranjeeta A., Cheng L., Kirby K., Krishna N. (2020). A low-cost smartphone controlled sensor based on image analysis for estimating whole-plant tissue nitrogen (N) content in floriculture crops. Comput. Electron. Agric..

[31] Hassanijalilian O., Igathinathane C., Doetkott C., Bajwa S., Nowatzki J., Esmaeili S.A.H. (2020). Chlorophyll estimation in soybean leaves inffield with smartphone digital imaging and machine learning. Comput. Electron. Agric..

[32] Chung S., Breshears L.E., Yoon J. (2018). Smartphone near infrared monitoring of plant stress. Comput. Electron. Agric..

[33] Tao M., Huang X., Liu C., Deng R., Liang K., Qi L. (2020). Smartphone-based detection of leaf color levels in rice plants. Comput. Electron. Agric..

[34] Danh D.N.H., Vincent P., Chi P., Rocio V., Khoa D., Christian N. (2020). Night-based hyperspectral imaging to study association of horticultural crop leaf reflectance and nutrient status. Comput. Electron. Agric..

[35] Liu B., Yue Y., Li R., Shen W., Wang K. (2014). Plant Leaf Chlorophyll Content Retrieval Based on a Field Imaging Spectroscopy System. Sensors.

[36] Pérez-Patricio M., Aguilar-González A., Camas-Anzueto J.L., Navarro N.A.M., Grajales-Coutiño R. (2018). An FPGA-based smart camera for accurate chlorophyll estimations. Int. J. Circuit Theory Appl..

[37] Brambilla M., Romano E., Buccheri M., Cutini M., Toscano P., Cacini S., Massa D., Ferri S., Monarca D., Fedrizzi M. (2020). Application of a low-cost RGB sensor to detect basil (*Ocimum basilicum* L.) nutritional status at pilot scale level. Precis. Agric..

[38] Ye X., Abe S., Zhang S., Yoshimura H. (2020). Hiroyuki. Rapid and non-destructive assessment of nutritional status in apple trees using a new smartphone-based wireless crop scanner system. Comput. Electron. Agric..

[39] Kangji L., Zhengdao S., Wenping X., Xu C., Hanping M., Gang T. (2020). A fast modeling and optimization scheme for greenhouse environmental system using proper orthogonal decomposition and multi-objective genetic algorithm. Comput. Electron. Agric..

[40] Chen X. Research on Data Interpolation Model with Missing Data for Intelligent Greenhouse Control. Proceedings of the 2019 International Conference on Robots & Intelligent System (ICRIS).

[41] Wu H., Li Q., Zhu H., Han X., Li Y., Yang B. (2020). Directional sensor placement in vegetable greenhouse for maximizing target coverage without occlusion. Wirel. Netw..

[42] Atefi A., Ge Y., Pitla S., Schnable J. (2019). In vivo human-like robotic phenotyping of leaf traits in maize and sorghum in greenhouse. Comput. Electron. Agric..

[43] Geng X., Zhang Q., Wei Q., Zhang T., Cai Y., Liang Y., Sun X. (2019). A Mobile Greenhouse Environment Monitoring System Based on the Internet of Things. IEEE Access.

[44] Ma D., Carpenter N., Maki H., Rehman T.U., Tuinstra M.R., Jin J. (2019). Greenhouse environment modeling and simulation for microclimate control. Comput. Electron. Agric..

[45] Uyeh D.D., Ramlan F.W., Mallipeddi R., Park T., Woo S., Kim J., Kim Y., Ha Y. (2019). Evolutionary Greenhouse Layout Optimization for Rapid and Safe Robot Navigation. IEEE Access.

[46] Nissimov S., Goldberger J., Alchanatis V. (2015). Obstacle detection in a greenhouse environment using the Kinect sensor. Comput. Electron. Agric..

[47] Bontsema J., van Henten E.J., Gieling T.H., Swinkels G.L.A.M. (2011). The effect of sensor errors on production and energy consumption in greenhouse horticulture. Comput. Electron. Agric..

[48] Pratim R.P. (2017). Internet of Things for Smart Agriculture: Technologies, Practices and Future Direction. J. Ambient. Intell. Smart Environ..

[49] Kinjal A.R., Patel B.S., Bhatt C.C. (2018). Smart Irrigation: Towards Next Generation Agriculture. Internet of Things and Big Data Analytics Toward Next-Generation Intelligence.

[50] Mishra B., Kertesz A. (2020). The Use of MQTT in M2M and IoT Systems: A Survey. IEEE Access.

[51] Dobrescu R., Merezeanu D., Mocanu S. (2019). Context-aware control and monitoring system with IoT and cloud support. Comput. Electron. Agric..

[52] Yang J., Liu M., Lu J., Miao Y., Hossain M.A., Alhamid M.F. (2018). Botanical Internet of Things: Toward Smart Indoor Farming by Connecting People, Plant, Data and Clouds. Mob. Netw. Appl..

[53] Akyildiz I.F., Su W., Sankarasubramaniam Y., Cayirci E. (2002). Wireless sensor networks: A survey. Comput. Netw..

[54] Zhou Y., Duan J. (2016). Design and Simulation of a Wireless Sensor Network Greenhouse-Monitoring System Based on 3G Network Communication. Int. J. Online Eng. (iJOE).

[55] Zhou Y., Xie Y., Shao L. (2016). Simulation of the Core Technology of a Greenhouse-Monitoring System Based on a Wireless Sensor Network. Int. J. Online Eng. (iJOE).

[56] Astillo P.V., Kim J., Sharma V., You I. (2020). SGF-MD: Behavior Rule Specification-Based Distributed Misbehavior Detection of Embedded IoT Devices in a Closed-Loop Smart Greenhouse Farming System. IEEE Access.

[57] Kocian A., Massa D., Cannazzaro S., Incrocci L., Milazzo P., Chessa S., Ceccanti C. (2020). Dynamic Bayesian network for crop growth prediction in greenhouses. Comput. Electron. Agric..

[58] Lekbangpong N., Muangprathub J., Srisawat T., Wanichsombat A. (2019). Precise Automation and Analysis of Environmental Factor Effecting on Growth of St. John’s Wort. IEEE Access.

[59] Chen M., Yang J., Zhu X., Wang X., Liu M., Song J. (2017). Smart Home 2.0: Innovative Smart Home System Powered by Botanical IoT and Emotion Detection. Mob. Netw. Appl..

[60] Kamilaris A., Prenafeta-Boldú F.X. (2018). Deep learning in agriculture: A survey. Comput. Electron. Agric..

[61] Liakos K., Busato P., Moshou D., Pearson S., Bochtis D. (2018). Machine Learning in Agriculture: A Review. Sensors.

[62] Yao C., Zhang Y., Zhang Y., Liu H. (2017). Application of Convolutial Neural Network in Classification of High Resolution Agricultural Remote Sensing Images. International Archives of the Photogrammetry, Remote Sensing and Spatial Information Sciences, Proceedings of the 2017 ISPRS Geospatial Week 2017, Wuhan, China, 18–22 September 2017.

[63] Boulent J., Foucher S., Théau J., St-Charles P. (2019). Convolutional Neural Networks for the Automatic Identification of Plant Diseases. Front. Plant Sci..

[64] Singh A.K., Ganapathysubramanian B., Sarkar S., Singh A. (2018). Deep Learning for Plant Stress Phenotyping: Trends and Future Perspectives. Trends Plant Sci..

[65] Zhang J., Zhu Y., Zhang X., Ye M., Yang J. (2018). Developing a Long Short-Term Memory (LSTM) based model for predicting water table depth in agricultural areas. J. Hydrol..

[66] An J., Li W., Li M., Cui S., Yue H. (2019). Identification and Classification of Maize Drought Stress Using Deep Convolutional Neural Network. Symmetry.

[67] Zhuang S., Wang P., Jiang B., Li M., Gong Z. (2017). Early detection of water stress in maize based on digital images. Comput. Electron. Agric..

[68] Zhu N., Liu X., Liu Z., Hu K., Wang Y., Tan J., Huang M., Zhu Q., Ji X., Jiang Y. (2018). Deep learning for smart agriculture: Concepts, tools, applications, and opportunities. Int. Agric. Biol. Eng..

[69] Mora-Fallas A., Goëau H., Joly A., Bonnet P., Mata-Montero E. (2020). Segmentación de instancias para detección automática de malezas y cultivos en campos de cultivo. Revista Tecnología En Marcha.

[70] Sa I., Ge Z., Dayoub F., Upcroft B., Perez T., McCool C. (2016). DeepFruits: A Fruit Detection System Using Deep Neural Networks. Sensors.

[71] Kang H., Chen C. (2019). Fruit Detection and Segmentation for Apple Harvesting Using Visual Sensor in Orchards. Sensors.

[72] Chen S.W., Shivakumar S.S., Dcunha S., Das J., Okon E., Qu C., Taylor J., Kumar V. (2017). Counting Apples and Oranges With Deep Learning: A Data-Driven Approach. IEEE Robot. Autom. Lett..

[73] Rodrigues E.R., Oliveira I., Cunha R., Netto M. DeepDownscale: A Deep Learning Strategy for High-Resolution Weather Forecast. Proceedings of the 2018 IEEE 14th International Conference on e-Science (e-Science).

[74] Wibisono M.N., Ahmad A.S. Weather forecasting using Knowledge Growing System (KGS). Proceedings of the 2017 2nd International Conferences on Information Technology, Information Systems and Electrical Engineering (ICITISEE).

[75] Rousseau D., Dee H., Pridmore T., Kumar J., Pratap A., Kumar S. (2015). Imaging Methods for Phenotyping of Plant Traits. Phenomics in Crop Plants: Trends, Options and Limitations.

[76] Kalaji H.M., Schansker G., Brestic M., Bussotti F., Calatayud A., Ferroni L., Goltsev V., Guidi L., Jajoo A., Li P. (2017). Frequently asked questions about chlorophyll fluorescence, the sequel. Photosynthesis Research.

[77] Made in China, Ningbo Peacefair Elevtronic Technology CO. LTD (2019). PZEM004T, Single Phase TTL Modbus Electric Power Meter. https://peacefair.en.made-in-china.com/product/zygxPIcSbuhV/China-Peacefair-Pzem-004t-Single-Phase-Ttl-Modbus-Electric-Power-Meter.html.

[78] Greene B.R., Segales A.R., Waugh S., Duthoit S., Chilson P.B. (2018). Considerations for temperature sensor placement on rotary-wing unmanned aircraft systems. Atmos. Meas. Tech..

[79] Barnett E., Gosselin C. (2015). Large-scale 3D printing with a cable-suspended robot. Addit. Manuf..

[80] (2018). Bosch-Sensortec, BME280 Combined Humidity and Pressure Sensor, Version 1.6 BST-BME280-DS002-15. https://www.bosch-sensortec.com/media/boschsensortec/downloads/datasheets/bst-bme280-ds002.pdf.

[81] (2020). DF-Robot, Gravity Analog Infrared CO_2_ Sensor for Arduino SKU SEN0219. https://www.dfrobot.com/product-1549.html.

[82] (2016). Sparkfun-Vishay Semiconductors VEML6075, Datasheet VEML6075, Document Number: 84304. https://cdn.sparkfun.com/assets/3/c/3/2/f/veml6075.pdf.

[83] (2019). Vishay Semiconductors VEML7700, Datasheet VEML7700, Document Number: 84286. https://www.vishay.com/docs/84286/veml7700.pdf.

[84] (2019). Seeed Studio the IoT Hardware Enabler, Groove Sensor, Groove Gas Sensor V2 (Multichannel). https://wiki.seeedstudio.com/Grove-Multichannel-Gas-Sensor-V2/.

[85] (2016). Raspberry PI, Accessories, PI NoIR Camera v2. https://www.raspberrypi.org/products/pi-noir-camera-v2/?resellerType=home.

[86] (2015). LEPRON FLIR, LWIR Micro Thermal Camera Module 2.5. https://lepton.flir.com/wp-content/uploads/2015/06/lepton-2pt5-datasheet-04195.pdf.

[87] (2019). DFRobot, Waterproof DS18B20 Digital Temperature Sensor for Arduino SEN-0198. https://www.dfrobot.com/product-689.html.

[88] (2019). DFRobot, Gravity: Analog TDS Sensor/Meter SEN-0244. https://www.dfrobot.com/product-1662.html.

[89] (2019). DFRobot, Gravity: Analog Spear Tip pH Sensor/Meter Kit SEN-0249. https://www.dfrobot.com/product-1668.html.

[90] (2019). DFRobot, Gravity: Analog pH Sensor/Meter Kit V2 SEN-0237A. https://www.dfrobot.com/product-1628.html.

[91] (2019). DFRobot, Gravity: Analog Turbidity Sensor For Arduino SEN-0189. https://www.dfrobot.com/product-1394.html.

[92] (2019). DFRobot, TCS3200 RGB Color Sensor for Arduino SEN-0101. https://www.dfrobot.com/product-540.html.

[93] (2019). DFRobot, TOF Sense Laser Ranging Sensor (5m) SEN-0337. https://www.dfrobot.com/product-2004.html.

[94] Li Y., Manoharan S. A performance comparison of SQL and NoSQL databases. Proceedings of the 2013 IEEE Pacific Rim Conference on Communications, Computers and Signal Processing (PACRIM).

[95] Cukrov M., Jerončić L., Prelogović L. (2017). Utjecaj Kontroliranog Vodnog Stresa na Sadržaj Bioaktivnih Spojeva u Hidroponskom Uzgoju Rikole (Eruca Sativa Mill.) i Špinata (Spinacia oleracea L.).

[96] He K., Zhang X., Ren S., Sun J. Deep Residual Learning for Image Recognition. Proceedings of the 2016 IEEE Conference on Computer Vision and Pattern Recognition (CVPR).

[97] Szegedy C., Ioffe S., Vanhoucke V., Alemi A. (2016). Inception-v4, Inception-ResNet and the Impact of Residual Connections on Learning. arXiv.

[98] Huang G., Liu Z., Maaten L.V.D., Weinberger K.Q. Densely Connected Convolutional Networks. Proceedings of the 2017 IEEE Conference on Computer Vision and Pattern Recognition (CVPR).

[99] Mingxing T., Quoc V.L. (2020). EfficientNet: Rethinking Model Scaling for Convolutional Neural Networks. arXiv.

[100] Marjani M., Nasaruddin F., Gani A., Karim A., Hashem I.A.T., Siddiqa A., Yaqoob I. (2017). Big IoT Data Analytics: Architecture, Opportunities, and Open Research Challenges. IEEE Access.

